# Personality Predictors of Emergency Department Post-Discharge Outcomes

**DOI:** 10.5964/ps.7193

**Published:** 2021-11-22

**Authors:** Olivia E. Atherton, Emily C. Willroth, Ted Schwaba, Ayla J. Goktan, Eileen K. Graham, David M. Condon, Mitesh B. Rao, Daniel K. Mroczek

**Affiliations:** 1 Department of Medical Social Sciences, Feinberg School of Medicine, Northwestern University, Chicago, IL, USA.; 2 Department of Psychology, University of Texas Austin, Austin, TX, USA.; 3 College of Education and Human Development, University of Louisville, Louisville, KY, USA.; 4 Department of Psychology, University of Oregon, Eugene, OR, USA.; 5 Department of Emergency Medicine, Stanford University, Palo Alto, CA, USA.; 6 Department of Psychology, Northwestern University, Evanston, IL, USA.

**Keywords:** personality traits, Big Five, emergency medicine, behavioral medicine, adherence, compliance, post-discharge outcomes, health behavior

## Abstract

Personality traits are important predictors of health behaviors, healthcare utilization, and health outcomes. However, we know little about the role of personality traits for emergency department outcomes. The present study used data from 200 patients (effective Ns range from 84 to 191), who were being discharged from the emergency department at an urban hospital, to investigate whether the Big Five personality traits were associated with post-discharge outcomes (i.e., filling prescriptions, following up with primary care physician, making an unscheduled return to the emergency department). Using logistic regression, we found few associations among the broad Big Five domains and post-discharge outcomes. However, results showed statistically significant associations between specific Big Five items (e.g., “responsible”) and the three post-discharge outcomes. This study demonstrates the feasibility of assessing personality traits in an emergency medicine setting and highlights the utility of having information about patients’ personality tendencies for predicting post-discharge compliance.

Emergency departments can be chaotic places. Blunt force trauma, stomach pain, mysterious bumps and rashes, alcohol poisoning, hypothermia – the ailments that bring people to emergency departments are infinite in scope, yet physicians and nurses are tasked with the same broader goals: 1) identify the source of the problem, and 2) create a treatment plan. Oftentimes, these treatment plans require patients to follow post-discharge instructions after they leave the emergency department such as filling prescriptions and/or making an appointment to follow-up with their primary care physician. Outside of the controlled hospital setting, we know very little about the factors that predict *who* adheres to their post-discharge instructions and who does not. The present study aims to fill some of these gaps by using data from patients admitted to a large, urban emergency department to investigate the role of personality traits for adherence to post-discharge instructions and unscheduled returns to the emergency department.

We define personality traits as falling into five broad domains: Extraversion (the tendency to be sociable, assertive, and lively); Agreeableness (the tendency to be warm, kind, and cooperative); Conscientiousness (the tendency to be self-controlled, organized, and responsible); Neuroticism (the tendency to worry, be moody, and lack self-confi-dence); and Openness to Experience (the tendency to be creative, adventurous, and open-minded; John & Soto, 2021). The Big Five personality traits predict important health outcomes such as health behaviors and healthcare utilization (Friedman, 2008; Hakulinen et al., 2015a; Hakulinen et al., 2015b; Jokela et al., 2018; Sutin et al., 2016), disease onset and progression (Friedman, 2008; Sutin et al., 2013; Weston et al., 2015), and mortality risk (Graham et al., 2017; Jokela et al., 2013; Mroczek et al., 2009; Turiano et al., 2015). However, little work has been conducted at the intersection of personality science and emergency medicine.

Of the work that has been done, most studies have examined the personality profiles of emergency medicine personnel (i.e., are the personality profiles of emergency medicine personnel different from those of personnel who work in other medical specialties?; Jordan et al., 2018). The remaining studies often focused on the personality characteristics of people who use (versus do not use) emergency healthcare services (Chapman et al., 2009; Friedman et al., 2013; Hajek et al., 2017), suggesting there are self-selection effects of *who* visits the emergency department to begin with. For example, prior work has shown that individuals who are more extraverted and less agreeable have an increased likelihood of visiting the emergency department and/or being hospitalized (Chapman et al., 2009; Hajek et al., 2017; but see Friedman et al., 2013). Moreover, some work has found that individuals who are less conscientious and more neurotic are more likely to use emergency department services, though these associations were not robust to the inclusion of socio-demographic covariates (Chapman et al., 2009).

To our knowledge, no prior work has examined whether patients’ personality tendencies are associated with their *adherence* to emergency department post-discharge instructions or their likelihood of making an unscheduled return to the emergency department. Prior work that is non-specific to the emergency setting has suggested that individuals who are higher in Conscientiousness are more likely to follow through with their doctor’s orders and take their medication as prescribed (Hill & Roberts, 2011; Molloy et al., 2014), whereas individuals who are higher in Neuroticism are less likely to take their medication as prescribed (Jerant et al., 2011). Taken together, we suspect that certain Big Five personality traits, like Conscientiousness and Neuroticism, may be important predictors of adherence to emergency department post-discharge instructions (i.e., filling prescriptions, following up with one’s primary care physician) and the likely-hood of making an unscheduled return to the emergency department within 72 hours of discharge.

Because the Big Five domains are broad, multi-faceted characterizations of personality tendencies, the present study also explored whether specific Big Five items, otherwise known as “nuances” (McCrae, 2015; Mõttus et al., 2017; Mõttus et al., 2020), were associated with each of the post-discharge outcomes. Prior work has suggested that facets and items within domains have divergent associations with health outcomes, sometimes nullifying the effect at the Big Five domain level (Hagger-Johnson & Whiteman, 2007; Weiss & Costa, 2005). In addition to capturing these divergent associations, exploring the associations between Big Five *items* and post-discharge outcomes necessarily provides a more mechanistic explanation of *why* personality traits are related to these outcomes (Mõttus et al., 2020), which has broader implications for how we might intervene to change personality tendencies and reduce the likelihood of non-adherence and readmission to the emergency department.

## The Present Study

The present exploratory study uses data collected in an emergency department, with a short-term longitudinal follow-up (1–2 weeks later) to examine how personality traits predict post-discharge outcomes. This research makes several unique contributions to the literature. First, the present study examined the feasibility of measuring personality traits in an emergency medicine context. Psychosocial data collection in emergency department settings poses numerous challenges, given that the people who are in the emergency department are likely to have fewer cognitive and emotional capacities for engaging in a research study with abstract psychological questions than the typical participant. Second, the present study incorporates a short-term longitudinal design, with a follow-up assessment of the patients 1–2 weeks after discharge. This permits an investigation of the outcomes that individuals experience even after they leave the emergency department and provides a better understanding of how we might predict the likelihood of individuals making unscheduled returns to the emergency department. Third, we administered a reliable and well-validated personality measure commensurate with those used in large national probability samples such as the Health and Retirement Study (Sonnega et al., 2014) and the Midlife in the United States Survey (Brim et al., 2004). Further, we examine the broad Big Five domains and specific Big Five items in relation to emergency department post-discharge outcomes. Last, to our knowledge this is the first study that aims to understand whether patients’ personality traits predict emergency department post-discharge outcomes. Thus, the present work not only has important theoretical implications for the predictive power of personality characteristics on health outcomes, but it also has many practical implications for how we might use information about patients’ personality tendencies to improve their individual health while also reducing the public health costs associated with individuals making repeated visits to emergency departments. We do not have any hypotheses; the present research was not pre-registered and is exploratory.

## Method

### Participants and Procedures

The present study used data collected from patients at a 57-bed Emergency Department (a verified Level 1 Trauma Center) at a major urban university hospital in the United States. The present study was granted approval by the Northwestern University Institutional Review Board (Protocol # STU00094889; Behavior Profiling in the Emergency Department [ED]), and data were subsequently collected from August 2014 to January 2015. To recruit participants, patients who were close to being discharged from the emergency department were asked to participate (the nurses on staff provided guidance to the interviewers about which patients would be best to approach). The patients who agreed to participate were administered a brief mental awareness and cognitive impairment inventory: the Short Portable Mental Status Questionnaire (SPMSQ; Pfeiffer, 1975). Sixty-five percent of those who were approached agreed to participate and had intact intellectual functioning or only mild intellectual impairment (0–4 errors out of 10 possible errors on the SPMSQ), resulting in a sample of 200 patients (58% female) that were diverse with respect to age, race, ethnicity, and education level.^1^ Participants ranged in age from 17 to 86 years (median = 39). Forty-six percent identified as White, non-Hispanic/Latino, 30% identified as Black/African-American, 15% identified as Hispanic/Latino, 4% identified as Asian, and 5% identified as multiracial. In terms of education level, 6% of participants had less than a high school degree, 14% were high school graduates, 60% had some college or a college degree, and 20% had more than a college degree. The median income ranged from $50,000 to $100,000 per year. Data were collected electronically via tablet-based questionnaires and included participants’ reports of demographic characteristics, health behaviors (e.g., smoking), Big Five personality traits, health literacy, chronic conditions, and healthcare utilization. We excluded three participants from analyses because one chose the same response option for all of the Big Five personality items, and two others were rated as having poor data quality by the interviewer(s) who conducted the assessments (85% of the participants had “good” data quality and 14% had “fair” data quality).

Approximately 1–2 weeks after being discharged from the emergency department, patients were contacted via telephone and queried on several aspects of their emergency department visit(s) including the reason for their visit(s), mode(s) of transport, and whether they made an unscheduled return to the emergency department since their initial visit. Additionally, participants reported on the extent to which they adhered to their initial post-discharge instructions (i.e., followed-up with their primary care physician, filled prescriptions), as well as their satisfaction with their emergency department experience(s). For a full list of items administered in the study protocol, please see the study codebook in Supplementary Materials. Of the 197 people included in the present analyses, 160 completed the follow-up telephone survey (81%). The effective Ns range from 84 to 191. The majority of participants indicated that they went to the emergency department on their initial visit because of symptoms such as pain, dizziness/fainting, headaches/migraines, dehydration, high blood pressure, or kidney stones/urinary problems (44% of sample). The remaining participants went to the emergency department because of an accident (24%), an infection, virus, or allergy (12%), women’s health issues related to pregnancy or miscarriage (6%), post-operative complications or doctor referrals (4%), a chronic condition (3%), psychiatric problems (3%), or something else (4%).

## Measures

### Big Five Personality Traits

At Time 1, participants completed the Midlife Development Inventory (MIDI) personality scales (Lachman & Weaver, 1997), a 26-item measure of the Big Five personality traits that has been shown to be a reliable and well-validated tool to measure personality (Brim et al., 2004; Sonnega et al., 2014). Based on prior research (Hill & Roberts, 2011), seven additional Conscientiousness items (i.e., industrious, traditional, self-controlled, persistent, orderly, reliable, impulsive) were also administered to participants because it was hypothesized at study conception that Conscientiousness had the most theoretical relevance for predicting post-discharge outcomes. Participants responded to the 33 items on a 4-point Likert scale ranging from 1 (*a lot*) to 4 (*not at all*). Items were reverse-scored (when appropriate) so that higher values represented greater levels of the personality trait and lower values represented lower levels of the personality trait. Items were then averaged together to create the Big Five personality domains: Extraversion (4 items, e.g., “outgoing”), Agreeableness (6 items, e.g., “caring”), Conscientiousness (12 items, e.g., “self-controlled”), Neuroticism (4 items, e.g., “worrying”), and Openness to Experience (7 items, e.g., “broadminded”).

### Post-Discharge Outcomes

At follow-up (1–2 weeks post-discharge), participants reported on three outcomes. Specifically, participants responded to dichotomous (0 = *no*, 1 = *yes*) questions that asked, “*Have you followed up with the physician(s) you were recommended to at discharge?*” and “*Did you fill the prescription(s) that you received at discharge?*” Participants also reported on whether they made an unscheduled return to an emergency department by responding to the following question: “*Have you returned to an Emergency Department since that discharge?*”

## Results

All analyses were conducted using R (R Core Team, 2021). Please see code for all analyses provided in Supplementary Materials. The data cannot be made publicly available on the OSF because: a) we did not receive IRB approval to share patients’ data, and b) the patients did not consent to having their deidentified data shared. Qualified researchers can email the corresponding author for access to a limited dataset to reproduce analyses. Analyses were not pre-registered; we report effect sizes, 95% confidence intervals, and exact *p*-values for all effects. For bivariate correlations and *t*-tests, we consider 95% confidence intervals that do not include 0 and *p*s < .05 as statistically significant. For the binary logistic regression models, we consider 95% confidence intervals that do not include 1 and *p* < .05 as statistically significant. We chose an alpha level of .05 for all analyses because even though the present study is exploratory, it has important practical implications for future psychosocial research in emergency medicine settings. We are cautious in our interpretations of *p*-values that approach .05, given that *p*-values closer to .05 are more likely to be false positives than smaller *p*-values (Colquhoun, 2017).

To investigate the potential impact of attrition, we compared individuals who did and did not participate in the follow-up survey on study variables assessed at Time 1 (i.e., Big Five personality traits, gender, age, income). Individuals who provided follow-up data were lower on Neuroticism at Time 1 than those who did not provide follow-up data (*M* = 2.19 vs. 2.51, *p* = .03, *d* = .44). There were no significant attrition differences by Extraversion, Agreeableness, Conscientiousness, Openness, age, gender, and income, all *p*s > .05.

To test whether there were selection effects, participants were asked at baseline: “*Besides this ED visit, have you been to the ED in the last 12 months?*” If selection effects are at play, participants who repeatedly go to the emergency department should show different levels of personality characteristics than participants who *had not* made another visit to the emergency department in the past 12 months. Sixty percent of the sample (114 out of 189) had *not* been to the emergency department in the past 12 months, and 40% of the sample (75 out of 189) had been to the emergency department in the past 12 months. We found that patients who had been to the emergency department in the past 12 months were lower on Conscientiousness (*M* = 3.16 vs. 3.28, *p* = .04, *d* = −.31), higher on Neuroticism (*M* = 2.39 vs. 2.16, *p* = .03, *d* = .33), and had lower incomes (*M* = 5.81 vs. 6.91, *p* = .02, *d* = −.39), compared to patients who had not been to the emergency department in the past 12 months.

Table 1 shows descriptive statistics and a bivariate intercorrelation matrix for all study variables. When queried about their post-discharge experiences, 60% of the sample (*n* = 97 out of 161) indicated that they followed up with their physician(s) as directed, whereas 21% (*n* = 34) did not follow up and 19% (*n* = 30) were not recommended to see their physician(s) post-discharge. Forty-eight percent of the sample (*n* = 77 out of 161) filled the prescription(s) they received at discharge, whereas 9% (*n* = 15) did not fill their prescriptions and 43% (*n* = 69) were not given a prescription by the Emergency Department. Last, 13% of the sample (*n* = 20 out of 160) returned to the emergency department within 72 hours of their initial visit, whereas 87% (*n* = 140) did not make an unscheduled return to the emergency department. The participants who indicated that they did not receive post-discharge instructions to fill a prescription or follow up with their primary care physician were treated as “missing” and not included in the regression analyses.

We also conducted follow-up analyses to determine whether there were any systematic differences among participants who did vs. did not receive instructions to fill a prescription or follow up with their primary care physician. 69 out of 161 people (43%) did not receive instructions to fill a prescription at discharge, whereas 92 out of 161 people (57%) did receive instructions to do so. 30 out of 161 people (19%) did not receive instructions to follow up with their primary care physician, whereas 131 out of 161 people (81%) did receive instructions to do so. There were no statistically significant differences between instruction vs. non-instruction groups on the Big Five personality traits or sociodemographic variables for either outcome, with the exception of income differences in those who received instructions to follow up with their primary care doctor. Participants who received instructions to follow up with their primary care physician had lower incomes than participants who did *not* receive these instructions.

Table 2 shows odds ratios and 95% confidence intervals for binary logistic regressions (with and without covariates) for the three post-discharge outcomes: filled prescription, followed-up with primary care physician, and made an unscheduled return to the emergency department. In the first set of analyses, we entered gender, age, income, and the Big Five domain scores as predictors of post-discharge outcomes. We included gender, age, and income as covariates because these factors have shown associations with both the independent (i.e., Big Five personality domains) and dependent variables (e.g., adherence, healthcare utilization) in prior research (e.g., Chapman et al., 2009); and thus, could confound the relationship between personality traits and post-discharge outcomes. In these analyses, we found that the demographic factors and the broad Big Five personality domains were not related to post-discharge outcomes, with one exception. Individuals with higher incomes were 1.18 times more likely to follow up with their primary care physician after being discharged from the emergency department than individuals who were less affluent. In unadjusted models without covariates, the aforementioned results hold and one additional effect emerged: Individuals higher in Extraversion were 2.76 times more likely to follow up with their primary care physician post-discharge.

**Figure 1:**
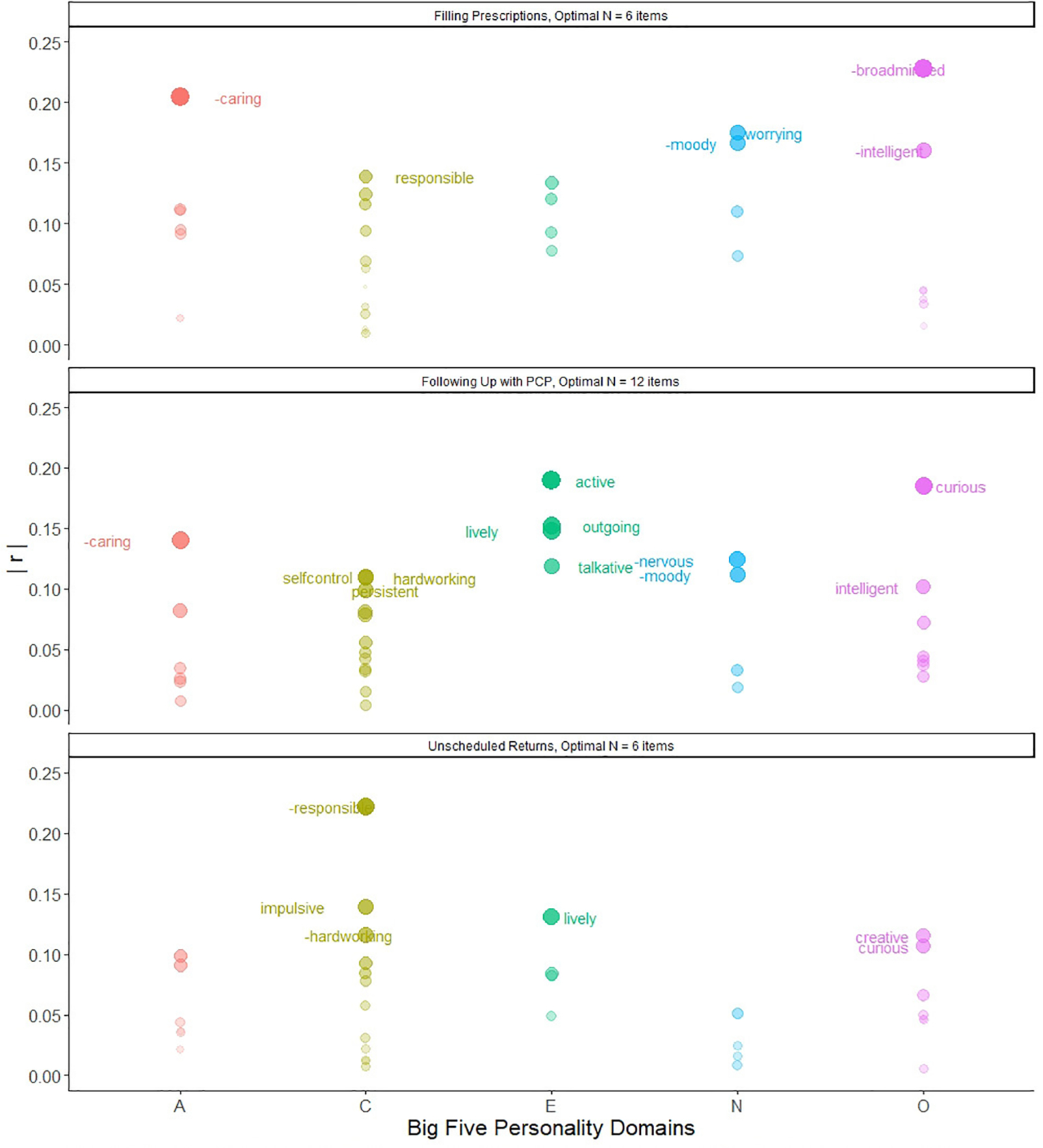
Big Five Items Predicting Post-Discharge Outcomes *Note.* A = Agreeableness; C = Conscientiousness; E = Extraversion; N = Neuroticism; O = Openness; | r | = absolute *r* values of item with criterion/outcome. Each dot represents one personality item. Size of dot tracks with size of *r* value. Transparency of dot indicates the proportion of replications (out of 1,000), with darker dots being more replicable and more transparent dots being less replicable. Dots with item labels are the best subset of item predictors of the outcome. Labels with a minus sign in front of the word indicate that the *r* association is negative.

We also conducted exploratory follow-up analyses to examine whether specific Big Five *items* were associated with each of the post-discharge outcomes. We used the bestScales function from the psych package (Elleman et al., 2020; Revelle, 2021), which identifies the items that are most correlated with a criterion (i.e., post-discharge out-comes) and then cross validates. We entered the 33 Big Five items as predictors of the three post-discharge outcomes (in separate models), with basic bootstrap aggregation (1,000 iterations); see Figure 1 for a visual depiction of the results and Tables S1–S3 in Supplementary Materials for the results in tabular form. The number of items that were likely to replicate in 50% or more of the bootstrapped replications ranged from 6 (for filling prescriptions and unscheduled returns) to 12 (for following up with one’s primary care physician).

## Discussion

### How Are Personality Traits Associated With Post-Discharge Outcomes?

When we examined whether the broad Big Five personality domains predicted post-dis-charge outcomes, we found no statistically significant associations. These results are somewhat surprising because prior work has shown that personality traits are related to medication and doctor adherence (Friedman et al., 2013; Hajek et al., 2017; Hill & Roberts, 2011; Jerant et al., 2011; Molloy et al., 2014), and we expected adherence to post-discharge instructions from the emergency department to be no exception. Although it is quite possible that the true associations are, in fact, null, there are several alternative explanations for why we may have observed this pattern of findings.

First, the sample size in the present study was small and thus, we are underpowered for observing what are likely to be small effects between broad personality traits and post-discharge outcomes. This is particularly true because not all participants indicated that the attending emergency medicine physician gave them post-discharge instructions to fill a prescription or follow up with their primary care physician, reducing sample sizes further (effective Ns ranging from 84 to 191).

Second, although there were no systematic personality differences between participants who did versus did not receive instructions to fill a prescription or follow up with their primary care physician, there are likely other selection biases at play that influence the effect size estimates. For example, when study participants were asked if they had been to the emergency department in the past 12 months (apart from their current visit), we found that there were systematic differences between individuals who *repeatedly* used the emergency department in the past year (60% of sample) and those who did not (40% of sample). Individuals who were lower in Conscientiousness, higher in Neuroticism, and from lower-income backgrounds were more likely to “self-select” into the emergency department setting to begin with, which is consistent with some prior work on the personality correlates of emergency department and hospital utilization (Chapman et al., 2009; Friedman et al., 2013; Hajek et al., 2017). Thus, by recruiting participants from the emergency department, our sample slightly over-represents individuals who have certain sociodemographic and personality characteristics. Systematic selection effects by sociodemographic and personality traits also highlight the need for future researchers to consider the complex interactions among sociodemographic, health literacy, healthcare access, and personality factors on adherence to post-discharge instructions and emergency department utilization.

Third, the Big Five domains are very broad and multi-faceted characterizations of personality; and thus, it is possible that components *within* each personality domain show divergent associations with post-discharge outcomes, nullifying the overall effects. To address this possibility, we explored whether specific personality items, or nuances, were related to the three post-discharge outcomes. From these item-level analyses, we gleaned information about the relative effect sizes of personality nuances (both within and across domains) with the three post-discharge outcomes, as well as some clues about *why* personality might be related to emergency department post-discharge outcomes (Mõttus et al., 2020). For example, there was notable variation in the magnitude of correlations among the 33 personality nuances and the three post-discharge outcomes, suggesting that there is added value in examining personality at this fine-grained level. Some components of the Big Five domains are more related to post-discharge outcomes than others. Furthermore, the “optimal” number of personality item predictors of the three outcomes varied from 6 items (for filling prescriptions and unscheduled returns) to 12 items (for following up with one’s primary care physician), suggesting that some post-discharge outcomes may be affected by a wider range of personality characteristics than others. Moreover, there were some personality nuances that were reliably associated with more than one outcome. Namely, people who were less caring (Agreeableness) and more moody (Neuroticism) were *less* likely to fill their prescriptions and follow up with their primary care physician, whereas people who were more responsible (Conscientiousness) were *more* likely to fill their prescriptions and *less* likely to make an unscheduled return to the emergency department. In addition, people who were more hardworking (Conscientiousness) were *more* likely to follow up with their primary care physician and *less* likely to make an unscheduled return to the emergency department. Yet, there were several instances where same-domain personality nuances were related to the same outcome in opposite directions (e.g., *higher* levels of worrying and *lower* levels of moodiness were associated with a higher likelihood of filling prescriptions), or were related to different outcomes in opposite directions (e.g., higher levels of liveliness and curiosity were positively associated with following up with one’s primary care physician *and* positively related to making an unscheduled return to the emergency department). Taken together, it seems as though digging beneath the surface of the broad Big Five domains can improve our understanding of how individual differences may predict emergency department post-discharge outcomes, though we need more highly-powered investigations of the associations between nuances and post-discharge outcomes before drawing any specific inferences.

### Considerations for Future Personality Research in the Emergency Medicine Setting

#### Sample Size

Although unique in its study design and population, the present study is limited by its rather small sample size (effective Ns range from 84 to 191). The intent of the present study was to be a proof-of-concept to serve as the basis for future federal grant applications at the intersection of personality science and emergency medicine. Due to the expense of conducting research in an emergency department setting, the present study is likely underpowered for detecting what are likely to be small associations among personality traits and compliance with post-discharge outcomes. Because of the small sample size, exploratory data analytic approach, and wide confidence intervals of effect sizes, all of the findings reported in this manuscript should be interpreted with caution until they are directly replicated with a larger sample. This is especially true for the item-level analyses where all 33 items are entered as predictors into the model. It will be necessary for future well-powered research to replicate the present findings, ideally with pre-registered analytic plans, to ascertain that the direction, magnitude, and importance of effect sizes are robust enough to warrant future application in medical settings.

#### Feasibility

A basic question that we were interested in addressing was the extent to which we could assess psychosocial constructs in the often chaotic emergency department setting. Patients visit the emergency department for a variety of reasons, but the immediacy of those reasons likely contributes to patients being more distracted and more burdened with cognitive, emotional, and physical fatigue than is typically seen in other research contexts. When we examined data quality in the present study, we found surprisingly few issues to contend with. Two-thirds of the patients who were approached in the emergency department agreed to participate and passed the brief cognitive status inventory. Moreover, out of the 200 people included in the sample, we only had to exclude two participants for poor data quality (as reported by the interviewers conducting the assessments) and one participant for non-differentiation of the personality item responses (i.e., straight-lining). In fact, the vast majority of the sample (85%) were rated as having good data quality by the interviewers.

Based on reliability analyses, we also found that the Big Five personality traits had high internal consistencies, with alpha reliabilities ranging from .70 (Conscientiousness) to .82 (Agreeableness). These reliability coefficients are comparable to, or even higher than, the alphas observed in other large-scale nationally-representative studies that used the same personality inventory, such as the Midlife in the United States Study (Brim et al., 2004), which shows alphas ranging from .58 (Conscientiousness) to .80 (Agreeableness). In sum, despite the fact that the patients in the present sample are likely experiencing more stress and cognitive, emotional, and physical load than the typical research participant, we found that we were able to obtain reliable signals of individual differences in personality even in this high-intensity setting, demonstrating the feasibility of collecting psychosocial patient data in this setting.

#### Representativeness of Sampling

Given the sensitive nature of collecting patient data in an emergency department setting, the interviewers for the present study worked in tandem with the nurses on staff, who provided advice about which patients might be amenable to approach, and alternately, which patients were in distress and likely would be unable to participate. Although this approach was a major boon to data collection, it is likely that this guidance from the nurses inadvertently biased the sample because perceived mood and illness burden are systematically related to personality traits, namely Conscientiousness and Neuroticism. Likewise, because many of the patients were close to being discharged from the emergency department, this is not necessarily a representative sample of all emergency department patients, given that the patients who came in with the most severe problems or least lucidity would not be represented in the participant pool. That being said, the conditions that bring people to the emergency department cut across important sociodemographic characteristics, bolstering sample heterogeneity. This is particularly true for the present study because the emergency department where patient data were collected was at a major, urban hospital that draws people from affluent neighborhoods in the immediate proximity as well as from low-income neighborhoods several miles away.

Nevertheless, data collection in this setting is complex because there needs to be a balance of respecting patients who are already under a significant amount of stress versus minimizing sample bias. We see two ways of dealing with this issue in future research: 1) half of the sample is recruited via tight collaboration between the research team and healthcare providers, whereas the other half of the sample is not recruited based on advice from the nurses, to allow for direct tests of sampling bias based on the key constructs of interest; and 2) collect data from someone other than the patient. In many cases, a close family member or friend accompanies a patient to the emergency department, and this close other may be a reliable source of information about the patient’s personality, are presumably under less distress than the patient themselves, and are less likely to feel burdened by study participation. Moreover, collecting informant-reports in the emergency department setting would help to reduce inflated effect sizes due to shared method variance, particularly if the patient provides self-reports at the follow-up visit(s).

#### Study Protocol

We have several recommendations for future research study protocols. First, we found it useful to look at both broad personality domains as well as specific personality nuances. Because both domain- and item-level approaches have advantages and disadvantages (in terms of reliability and the capacity to provide mechanistic explanations), reporting both in the same paper provides the fullest understanding of a particular phenomenon. This approach also has practical utility for future research and application. If future well-powered research replicates the predictive utility of *specific* personality items, then shorter personality inventories based on these items can be administered to patients, reducing participant burden. In addition, if there is a certain subset of items that are consistently associated with post-discharge outcomes, then future researchers and emergency medicine personnel will be able to more easily use this information to identify the patients most at risk for unfavorable outcomes and tailor their post-discharge treatment plan to suit their personality tendencies (e.g., text message reminders to pick up prescriptions or follow up with a primary care physician).

Second, we recommend future researchers add several variables to the study protocol: insurance (i.e., private, public, underinsured, uninsured); more context for the reason of the visit (e.g., chronic condition) and probability of reoccurrence (i.e., how many times the patient has been to the emergency department for the same reason(s)); a wider range of post-discharge instructions beyond prescriptions and following up with one’s primary care physician; additional outcomes of an emergency department visit (e.g., symptom remission); the extent to which patients had difficulties complying with administered instructions (e.g., “to what extent did you experience difficulties carrying out the instructions given to you at discharge?”) and *why* they experienced difficulty (in a standardized format across participants).

Third, the time lag between the initial assessment and follow-up was short, with participants queried approximately 1–2 weeks after their initial visit to the emergency department. Thus, it is possible that some participants filled their prescriptions, followed up with their primary care physician, and/or made an unscheduled return to the emergency department 15+ days after their initial visit, though these outcomes are not considered in the data collected in the present study. Future research would benefit from conducting multiple assessments of varying time lengths post-discharge, in order to better understand the long-term influence of personality traits on post-discharge outcomes. Similarly, assessing personality traits and health at multiple timepoints would help researchers to draw stronger, *directional* inferences about whether personality traits have an influence on post-discharge outcomes above and beyond prior levels, as well as whether there are influences of repeatedly visiting an emergency department on subsequent personality tendencies.

## Conclusion

Upon further examinations of the replicability and generalizability of the present findings with highly-powered replication studies, it may become clear that individual differences in personality are one way to identify patients who are most at risk for not adhering to post-discharge instructions and making repeated visits to the emergency department. If personality traits can help us to identify the patients most at risk for these unfavorable outcomes, then future researchers and emergency medicine personnel can work together to develop systems that utilize knowledge about patients’ personality to improve individual health and reduce public health costs associated with repeated visits to the emergency department.

## Supplementary Material

SOM

## Figures and Tables

**Table 1:** Means, Standard Deviations, and Correlations Among Study Variables

Variable	*M*	*SD*	*α*	1.	2.	3.	4.	5.	6.	7.	8.	9.	10.
1. Gender	0.58	0.50	-										
2. Age	41.48	15.76	-	−.17* [−.30, −.03]									
3. Income	6.49	2.88	-	−.06 [−.21, .09]	.09 [−.06, .24]								
4. Extraversión	3.39	0.57	.73	.08 [−.07, .22]	−.06 [−.20, .09]	−.09 [−.23, .07]							
5. Agreeableness	3.59	0.46	.82	.24* [.10, .37]	−.01 [−.15, .14]	−.14 [−.29, .01]	.50* [.39, .60]						
6. Conscientiousness	3.24	0.39	.71	.00 [−.14, .15]	.14* [.00, .28]	.18* [.03, .33]	.30* [.16, .42]	.37* [.24, .48]					
7. Neuroticism	2.25	0.71	.72	.10 [−.05, .24]	−.20* [−.33, −.05]	.02 [−.13, .17]	−.24* [−.37, −.10]	−.24* [−.37, −.10]	−.24* [−.37, −.10]				
8. Openness	3.39	0.45	.73	−.01 [−.15, .13]	.03 [−.12, .17]	−.11 [−.26, .04]	.55* [.44, .64]	.42* [.29, .53]	.29* [.16, .42]	−.20* [−.33, −.06]			
9. Filling Prescription	0.84	0.37	-	−.03 [−.23, .17]	.07 [−.14, .27]	.07 [−.15, .28]	.02 [−.19, .22]	.00 [−.20, .21]	.02 [−.18, .23]	−.03 [−.23, .18]	−.06 [−.26, .14]		
10. Follow-Up with PCP	0.74	0.44	-	.04 [−.13, .21]	.16 [−.01, .32]	.22* [.04, .38]	.19* [.02, .35]	.00 [−.17, .17]	.08 [−.09, .25]	−.10 [−.27, .07]	.05 [−.12, .22]	.20 [−.02, .40]	
11. Unscheduled Return to ED	0.12	0.33	-	−.03 [−.18, .13]	.06 [−.10, .22]	−.09 [−.25, .08]	.07 [−.08, .23]	.04 [−.11, .20]	.03 [−.13, .18]	−.02 [−.17, .14]	.06 [−.10, .21]	.09 [−.12, .29]	.16 [−.02, .32]

*Note. M* = mean; *SD* = standard deviation; α = alpha reliability; PCP = primary care physician; ED = emergency department. Values in square brackets indicate the 95% confidence interval for each correlation. Effective Ns range from 84 to 191, depending on the pairwise correlation.

* *p* < .05

**Table 2:** Odds Ratios from Binary Logistic Regressions Between the Big Five and Post-Discharge Outcomes With and Without Covariates

	Filling prescription post-discharge		Following-up with primary care physician post-discharge		Unscheduled return to the emergency department	
Variable	*N* = 84	*N* = 92	*N* = 116	*N* = 131	*N* = 142	*N* = 160
Gender	1.20 [0.32, 4.46]		0.56 [0.20, 1.54]		1.50 [0.49, 4.60]	
Age	1.02 [0.97, 1.06]		1.03 [0.99, 1.06]		1.02 [0.99, 1.05]	
Income	1.05 [0.84, 1.33]		1.18* [1.01, 1.39]		0.87 [0.72, 1.05]	
Extraversion	1.12 [0.27, 4.59]	1.46 [0.38, 5.77]	2.55 [0.91, 7.16]	2.76* [1.09, 7.39]	1.72 [0.52, 5.64]	1.41 [−0.00, 4.38]
Agreeableness	1.73 [0.35, 8.49]	0.99 [0.23, 3.81]	0.73 [0.20, 2.62]	0.55 [0.17, 1.60]	0.81 [0.17, 3.94]	1.03 [−0.00, 4.41]
Conscientiousness	1.23 [0.21, 7.30]	1.21 [0.25, 5.76]	0.86 [0.22, 3.41]	1.15 [0.35, 3.73]	1.60 [0.32, 8.11]	0.99 [−0.00, 3.98]
Neuroticism	1.14 [0.47, 2.78]	0.90 [0.40, 2.03]	0.84 [0.41, 1.73]	0.82 [0.45, 1.50]	1.16 [0.52, 2.59]	1.01 [−0.00, 2.01]
Openness	0.43 [0.08, 2.45]	0.45 [0.07, 2.32]	0.88 [0.27, 2.87]	0.72 [0.24, 2.13]	0.65 [0.16, 2.62]	1.16 [−0.00, 4.49]

*Note.* Listed Ns are the number of cases that have complete data in the binary logistic regression models with and without covariates. Values in the tables are odds ratios with 95% confidence intervals in brackets.

* *p* < .05.

## Relevance Statement

Researchers and physicians may be able to use information about patients’ personality to identify those most at risk for unfavorable post-discharge outcomes and tailor their treatment plans to suit their personality tendencies to improve health.

## Key Insights

- Personality traits can be reliably assessed in emergency department setting.
- No associations among broad Big Five traits and post-discharge compliance.
- Several associations among specific Big Five items and post-discharge compliance.

## Footnotes

1) There are no prior publications with this dataset to report.
