# Supplementary material for: Personality Predictors of Emergency Department Post-Discharge Outcomes
Source: Personal Sci. Author manuscript; Available in PMC 2022 Mar 29. (PMC8963191; doi:10.5964/ps.7193)
Supplement: SOM [file NIHMS1787680-supplement-SOM.docx]

**Supplemental Material**

| Table S1 | | | |
| --- | --- | --- | --- |
| *Big Five Items Predicting Filling Prescriptions* | | | |
|  | Frequency of Replications | Mean (r) | SD (r) |
| **broadminded** | **906** | **-0.23** | **0.06** |
| **caring** | **905** | **-0.2** | **0.04** |
| **worrying** | **631** | **0.17** | **0.12** |
| **moody** | **586** | **-0.17** | **0.11** |
| **intelligent** | **566** | **-0.16** | **0.08** |
| **responsible** | **488** | **0.14** | **0.12** |
| lively | 414 | -0.13 | 0.08 |
| industrious | 422 | -0.12 | 0.09 |
| outgoing | 420 | -0.12 | 0.1 |
| thorough | 394 | -0.12 | 0.09 |
| helpful | 306 | -0.11 | 0.07 |
| friendly | 360 | -0.11 | 0.09 |
| calm | 382 | 0.11 | 0.11 |
| softhearted | 308 | 0.1 | 0.1 |
| impulsive | 292 | 0.09 | 0.1 |
| active | 354 | 0.09 | 0.12 |
| sympathetic | 330 | 0.09 | 0.11 |
| talkative | 287 | 0.08 | 0.12 |
| nervous | 299 | 0.07 | 0.11 |
| careless | 292 | 0.07 | 0.12 |
| traditional | 201 | 0.06 | 0.09 |
| hardworking | 72 | -0.05 | 0.07 |
| creative | 177 | -0.04 | 0.1 |
| adventurous | 149 | -0.04 | 0.1 |
| sophisticated | 167 | 0.04 | 0.1 |
| imaginative | 180 | 0.03 | 0.11 |
| persistent | 176 | -0.03 | 0.11 |
| orderly | 177 | -0.03 | 0.11 |
| warm | 221 | 0.02 | 0.13 |
| curious | 125 | -0.02 | 0.1 |
| reliable | 113 | 0.01 | 0.1 |
| organized | 196 | 0.01 | 0.12 |
| self-control | 106 | -0.01 | 0.09 |
| *Note*. Optimal Weighted Final N = 6 items (in bold). We used the bestScales function from the psych package, which identifies the Best Items Scales that are Cross validated, Unit weighted, Informative, and Transparent (BISCUIT). Frequency of replications is out of 1,000 basic bootstrap aggregations. | | | |

| Table S2 | | | |
| --- | --- | --- | --- |
| *Big Five Items Predicting Following Up with Primary Care Physician* | | | |
|  | Frequency of Replications | Mean (r) | SD (r) |
| **active** | **771** | **0.19** | **0.1** |
| **curious** | **747** | **0.19** | **0.09** |
| **outgoing** | **625** | **0.15** | **0.09** |
| **lively** | **608** | **0.15** | **0.09** |
| **caring** | **613** | **-0.14** | **0.06** |
| **nervous** | **501** | **-0.12** | **0.09** |
| **talkative** | **476** | **0.12** | **0.09** |
| **moody** | **462** | **-0.11** | **0.1** |
| **self-control** | **453** | **0.11** | **0.1** |
| **hardworking** | **450** | **0.11** | **0.1** |
| **intelligent** | **407** | **0.1** | **0.09** |
| **persistent** | **410** | **0.1** | **0.1** |
| softhearted | 319 | 0.08 | 0.09 |
| industrious | 297 | 0.08 | 0.08 |
| responsible | 297 | 0.08 | 0.09 |
| creative | 309 | 0.07 | 0.1 |
| careless | 264 | -0.06 | 0.09 |
| thorough | 205 | -0.05 | 0.08 |
| sophisticated | 221 | 0.04 | 0.09 |
| reliable | 175 | 0.04 | 0.09 |
| adventurous | 209 | -0.04 | 0.09 |
| broadminded | 179 | -0.04 | 0.08 |
| sympathetic | 198 | 0.03 | 0.09 |
| impulsive | 171 | -0.03 | 0.08 |
| worrying | 173 | -0.03 | 0.09 |
| organized | 179 | 0.03 | 0.09 |
| imaginative | 226 | -0.03 | 0.1 |
| helpful | 214 | 0.03 | 0.1 |
| warm | 202 | 0.02 | 0.1 |
| calm | 153 | -0.02 | 0.08 |
| traditional | 189 | -0.02 | 0.09 |
| friendly | 146 | 0.01 | 0.09 |
| orderly | 152 | 0 | 0.09 |
| *Note*. Optimal Weighted Final N = 12 items (in bold). We used the bestScales function from the psych package, which identifies the Best Items Scales that are Cross validated, Unit weighted, Informative, and Transparent (BISCUIT). Frequency of replications is out of 1,000 basic bootstrap aggregations. | | | |

| Table S3 | | | |
| --- | --- | --- | --- |
| *Big Five Items Predicting Unscheduled Returns to ED* | | | |
|  | Frequency of Replications | Mean (r) | SD (r) |
| **responsible** | **839** | **-0.22** | **0.11** |
| **impulsive** | **639** | **0.14** | **0.08** |
| **lively** | **694** | **0.13** | **0.07** |
| **hardworking** | **523** | **-0.12** | **0.1** |
| **creative** | **563** | **0.12** | **0.07** |
| **curious** | **498** | **0.11** | **0.07** |
| sympathetic | 471 | 0.1 | 0.07 |
| traditional | 418 | 0.09 | 0.08 |
| softhearted | 446 | 0.09 | 0.08 |
| thorough | 369 | 0.08 | 0.07 |
| outgoing | 400 | 0.08 | 0.08 |
| talkative | 360 | 0.08 | 0.07 |
| self-control | 325 | 0.08 | 0.07 |
| sophisticated | 338 | 0.07 | 0.09 |
| organized | 289 | -0.06 | 0.08 |
| moody | 289 | -0.05 | 0.09 |
| broadminded | 260 | 0.05 | 0.08 |
| active | 244 | 0.05 | 0.08 |
| imaginative | 218 | 0.05 | 0.08 |
| adventurous | 168 | 0.05 | 0.07 |
| helpful | 221 | 0.04 | 0.08 |
| friendly | 127 | 0.04 | 0.07 |
| warm | 197 | 0.04 | 0.08 |
| persistent | 240 | -0.03 | 0.09 |
| worrying | 210 | 0.02 | 0.08 |
| reliable | 180 | -0.02 | 0.08 |
| caring | 226 | -0.02 | 0.09 |
| calm | 242 | 0.02 | 0.09 |
| industrious | 220 | 0.01 | 0.09 |
| orderly | 169 | 0.01 | 0.08 |
| nervous | 147 | -0.01 | 0.08 |
| careless | 230 | 0.01 | 0.09 |
| intelligent | 241 | -0.01 | 0.09 |
| *Note*. Optimal Weighted Final N = 6 items (in bold). We used the bestScales function from the psych package, which identifies the Best Items Scales that are Cross validated, Unit weighted, Informative, and Transparent (BISCUIT). Frequency of replications is out of 1,000 basic bootstrap aggregations. | | | |
